# Supplementary material for: Spatial distribution of mixed milk feeding and its determinants among mothers of infants aged under 6 months in Ethiopia: Spatial and geographical weighted regression analysis
Source: PLoS One. 2025 Mar 5;20(3):e0317089. doi: 10.1371/journal.pone.0317089 (PMC11882095; doi:10.1371/journal.pone.0317089)
Supplement: Attachment — Submitted filename: CC. BY 4.0.docx [file pone.0317089.s002.docx]

**Date 26 September 2024**

**Subject: Response to Data Availability Statement Request for Manuscript PONE-D-24-28246R1**

**Dear Editor,**

**I hope this message finds you well.**

I am writing to submit my response regarding the Data Availability statement for my manuscript titled "Spatial distribution of mixed milk feeding and its determinants among mothers of infants aged under 6 months in Ethiopia: spatial and geographical weighted regression analysis" (Manuscript ID: PONE-D-24-28246R1).

Thank you for your continued assistance throughout this process.

Kind regards,

Mr. Mekuriaw Nibret Aweke

**Correspondent Author**

**Point by point response to the Academic editor and reviewer’s comments**

| 1. **Comments from the academic editor and responses** | |
| --- | --- |
| **Academic editor’s comments** | **Authors’ responses** |
| 1. We require you to either (1) present written permission from the copyright holder to publish these figures specifically under the CC BY 4.0 license, or (2) remove the figures from your submission:  a. You may seek permission from the original copyright holder of Figures 4, 5,6,8 and 9 to publish the content specifically under the CC BY 4.0 license. | **Dear editor,**  Thank you for your comments regarding the figures in my submission. I would like to clarify that the data source for Figures 4, 5, 6, 8, and 9 is the Central Statistical Agency (CSA) of Ethiopia. The CSA permits the following under the terms of their data use:  Share — copy and redistribute the material in any medium or format for any purpose, even commercially.  Adapt — remix, transform, and build upon the material for any purpose, even commercially.  These permissions align with the CC BY 4.0 license, which allows for sharing and adaptation as long as proper attribution is given. We have clearly indicated the source (CSA Ethiopia) in the figures, ensuring compliance with the license terms.  For further verification, you can review the terms of the CC BY 4.0 license at <https://creativecommons.org/licenses/by/4.0/>  Please let me know if any further clarification is needed |
| Please confirm whether this information is accurate, and we will update your Data Availability statement accordingly on your behalf. | **Dear editor,**  Thank you for providing the proposed Data Availability statement. I confirm that the information is accurate and aligns with the access process for the Ethiopian Mini Demographic and Health Survey (miniDHS) 2019 data. Please proceed with updating the Data Availability statement as outlined. |
