# Supplementary material for: Spatial distribution of mixed milk feeding and its determinants among mothers of infants aged under 6 months in Ethiopia: Spatial and geographical weighted regression analysis
Source: PLoS One. 2025 Mar 5;20(3):e0317089. doi: 10.1371/journal.pone.0317089 (PMC11882095; doi:10.1371/journal.pone.0317089)
Supplement: Attachment — Submitted filename: comment.docx [file pone.0317089.s003.docx]

**Comments to the Author:**
Appears that comments have been addressed.  Could benefit from some additional grammar editing.
