# Supplementary material for: Spatial distribution of mixed milk feeding and its determinants among mothers of infants aged under 6 months in Ethiopia: Spatial and geographical weighted regression analysis
Source: PLoS One. 2025 Mar 5;20(3):e0317089. doi: 10.1371/journal.pone.0317089 (PMC11882095; doi:10.1371/journal.pone.0317089)
Supplement: Attachment — Submitted filename: Reviewer comments.docx [file pone.0317089.s001.docx]

**Title: “Spatial distribution of mixed milk feeding and its determinants among mothers of infants aged under 6 months in Ethiopia: spatial and geographical weighted regression analysis”**

Here is an in-depth report addressing each of the checklist items and providing specific sections where issues have been identified:

**Title and Abstract:**

1. **(a)** The title is well crafted.

**(b)** In the **ABSTRACT:**

- **Method Section**: You need to clearly describe the total weighted sample of infants used in your study.
- **Result Section**: Include the weighted prevalence of Mixed Milk Feeding (MMF) in Ethiopia. Additionally, it's recommended to include numerical results such as the global Moran's I with p-value, the percentage of the maximum cluster size per population, etc.
- **Keywords**: Why are the keywords missing? Please include them in your abstract and arrange them according to scientific standards.
- Overall: The **abstract** does not effectively convey what was done in the study, so revisions are needed for clarity and completeness.

**INTRODUCTION:**

2. The introduction section presents the following issues:

- **In paragraph 5:** “Studies have shown that breastfeeding improves the emotional and psychological connection between mother and infant.” What are the studies? Lacks a relevant litrature.
- **In paragraph 9:** “To this day, various research has been carried out in Ethiopia to evaluate the exclusive breast feeding among children, including the bottle feeding.” Please put the relevance various research reference to establish the rationale.
- Overall, the introduction is well written.

**METHODS AND MATERIALS:**

3. **Data source:** The description of the setting, and locations is correctly provided. I think this is a secondary data analysis, the absence of eligibility criteria and participant selection details is acceptable so I didn’t see such thing in detail.
4. **Sampling procedure and population**: Do you believe that 'all living infants under 6 months of age' amounts to only 524 (based on the 2019 EMDHS)? If not, how was the sample of 524 infants selected? Please provide a clear description of the selection procedure.
5. **Data management and analysis**: Please put the version of STATA. “We remove missing values from our analysis by using the STATA drop command in combination with a logical or conditional statement.” But you mentioned “Missing data were managed based on the DHS guideline” under **data source** section. How do you relate those two sentences? Do you believe that “Removing” is considered as the management of missing data analysis? If not, mange your missing data based on imputation or other handling method.
6. Please thoroughly document all methods under the **'Spatial Scan Statistics'** and **'Spatial Regression'** sections, including relevant supporting references.

**RESULTS:**

7. Please format the **table title** clearly (e.g., 'Problem, Population, Area, 2024, n=') and ensure the **tables** are cited throughout the document with cross-references.
8. **Prevalence of mixed milk feeding in Ethiopia:”** In this study, the overall prevalence of MMF was 10.12% (95% CI 7.8, 13.01), with regional variations observed across Ethiopia”. The results were not displayed in any tables or graphs. Please provide the prevalence data in either a table or a graph, and reference any tables used.

**DISCUSSION:**

9. The '**Discussion**' section can be generally divided into 3 separate paragraphs. 1) Introductory paragraph/rationale of the study, 2) Intermediate paragraphs/compare and contrast with the most recent and relevant literature, 3) Concluding paragraph/indicating future directions. Then address such things as well.
10. While the manuscript mentions the study utilized nationally representative data as a strength indicating there is adequate power to detect the true effect of exposures. However, it does not provide a power calculation to support this claim.

**Recommendation**

This comprehensive report outlines the issues identified throughout the manuscript, with specific sections and requires substantial minor revisions to address these concerns and enhance the clarity, relevance, and presentation of results. After making the necessary minor revisions with re-write of manuscript, a re-evaluation is recommended for considering publication.
